# A new 915 MHz coaxial-line-based microwave plasma source

**DOI:** 10.1038/s41598-024-66455-6

**Published:** 2024-07-05

**Authors:** Robert Miotk, Jerzy Mizeraczyk, Mariusz Jasiński

**Affiliations:** 1 Institute of Fluid Flow Machinery, Polish Academy of Sciences, Fiszera 14, 80-231 Gdańsk, Poland; 2 Department of Marine Electronics, Gdynia Maritime University, Morska 81-87, 81-225 Gdynia, Poland

**Keywords:** Optical spectroscopy, Electrical and electronic engineering, Devices for energy harvesting

## Abstract

Microwave plasma is known for its versatility in providing tailored operating conditions (pressure, working gas composition and residence time of reagents) for specific applications. Microwave plasma sources (MPSs) are vital in modern applications, demanding continuous improvement. This work introduces a coaxial-line-based nozzleless MPS that operates at atmospheric pressure at an unique frequency of 915 MHz. The measured electrodynamic characteristics in nitrogen of the MPS highlighted the need for improved energy efficiency of the device. The main novelty of this work lies in improving an energy efficiency of the presented MPS, which led to an advanced new version of the device. To achieve this, a dual strategy is employed. Firstly, numerical simulations are used to design a construction modifications to the MPS, which should increase the efficiency of transferring microwave energy from the microwave source to the generated plasma. In this step, a standard model for homogeneous plasma and a two-port equivalent method were used. Then, the theoretical results were experimentally validated by manufacturing a new energy improved version of the MPS. In the new MPS the achieved reflected microwave power (losses) was less than 3% of incident microwave power in the tested range of nitrogen flow rate (50–100 Nl/min). Compared to the MPS before improvement, this means a two-fold decreasing the reflected microwave power. To test the new MPS, the electrodynamic characteristics of the new device version and properties of the microwave plasma generated in nitrogen, using optical emission spectroscopy (OES), were investigated. The OES was used to determine the vibrational *T*_vib_ and rotational *T*_rot_ temperatures of nitrogen molecules and molecular ions. In this work, the estimated *T*_vib_ and *T*_rot_ temperatures for nitrogen molecules ranged from 4000 to 5300 K, depending on discharge conditions, while for nitrogen molecular ions, the temperatures changed between 4700 and 6100 K, respectively. Both the *T*_vib_ and *T*_rot_ temperatures decrease linearly along the plasma flame.

## Introduction

Operating at atmospheric pressure has the advantage of eliminating the need for vacuum systems, allowing for direct interaction with the plasma. This makes atmospheric pressure microwave plasma sources (MPSs)^1^ suitable for a wide range of applications. One significant application is in the field of material processing, specifically for surface modification and film deposition^2–6^. The controlled release of reactive species from the plasma enables precise and efficient treatment of materials, resulting in improved surface properties, including heightened hardness, enhanced wear resistance, and superior corrosion protection.

Another important industrial sector where microwave plasma is employed is the chemical industry. The microwave plasma enables the synthesis of complex chemical compounds by providing a highly energetic and controllable environment for chemical reactions^7–10^. This offers several advantages, such as accelerated reaction rates, enhanced selectivity and reduced energy consumption when compared to conventional processes. Microwave plasma also plays an important role in environmental applications. It is a valuable tool for removing pollutants from industrial exhaust gases, decomposing harmful compounds and converting them into harmless substances. It is also used in waste treatment, enabling the disposal and treatment of hazardous waste materials through efficient and environmentally friendly processes. Moreover, microwave plasma technology shows potential in the field of energy production due to its high efficiency, low energy consumption, and controllable reaction environment. It can be used for the conversion of natural gas, biogas or other hydrocarbon gases into the production of gaseous fuels rich in hydrogen^11–13^. Additionally, it proves to be an efficient method for hydrogen production from liquid hydrogen carriers, such as alcohols^14–18^.

The MPSs are under constant pressure to improve their performance due to the evolving demands of modern applications. Thorough investigation and understanding of the properties of the generated plasma are crucial to meet industrial requirements. Additionally, the industry also focuses on parameters such as energy efficiency, process uniformity, and process cycle control. Achieving cost-effective microwave discharges is crucial for the practical application of microwave plasma in industry. Improving the construction of the MPS can lead to a reduction in the cost of the generated discharge.

The research presented in this work examines the properties of a coaxial-line-based nozzleless MPS. The presented MPS operates under atmospheric pressure conditions at unique frequency of 915 MHz, which is a much less common method of generating microwave discharges than 2.45 GHz. The main advantages of using 915 MHz compared to 2.45 GHz microwaves lie in its deeper penetration depth in the plasma and higher power capabilities. This advantages results in more efficient heating and processing of plasmo-chemical reactions, which is advantageous for applications requiring uniform energy distribution throughout the plasma volume. Additionally, the larger size of 915 MHz equipment allows for higher power capabilities, making it suitable for industrial applications requiring higher power levels.

**Figure 1:**
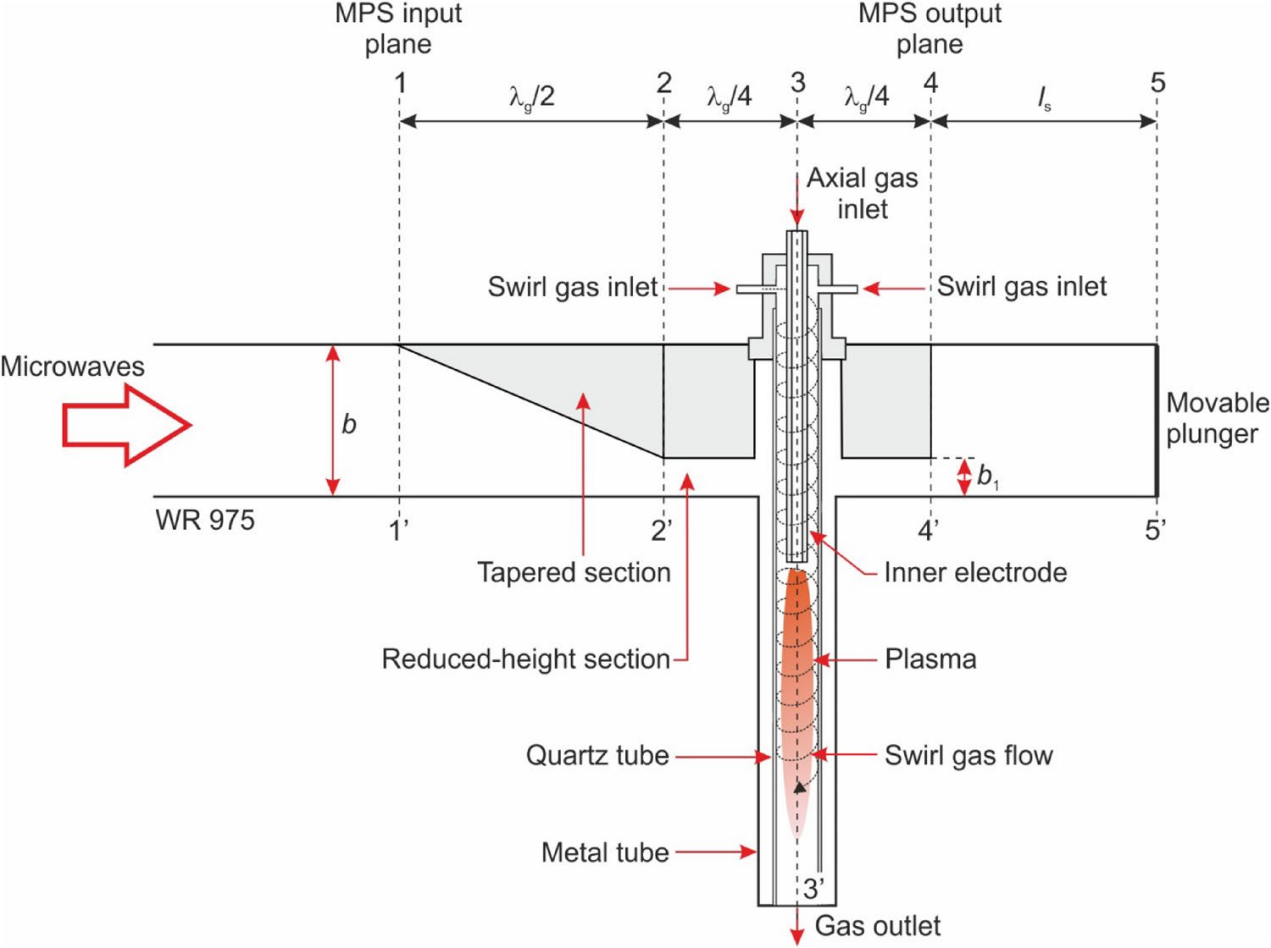
Sketch of the waveguide-supplied coaxial-line-based nozzleless MPS.

As part of the work, the electrodynamic characteristics of the MPS were measured. The electrodynamic characteristics of the MPS is the dependence of the *P*_R_/*P*_I_ ratio (the reflected *P*_R_/incident *P*_I_ microwave power in the waveguide) on the position of movable plunger *l*_S_ (see Fig. 1). The *P*_R_/*P*_I_ (*l*_S_) relations serve as an indicator of the MPS energy efficiency, specifically measuring the plasma’s effectiveness in absorbing microwave power. The microwave power *P*_A_ absorbed by the plasma is defined as the difference between the power *P*_I_ and the power *P*_R_, *P*_A_ = *P*_I_ − *P*_R_. Minimising the *P*_R_/*P*_I_ ratio is equal to maximising the *P*_A_ power absorbed by the generated plasma. The measured characteristics indicated a need to improve the energy efficiency of the MPS. Improving the construction of the MPS was crucial for achieving energy efficient and stable plasma generation.

The main novelty of this work lies in improving an energy efficiency of the presented MPS, which led to the advanced new version of the device. The electrodynamic properties of this new MPS and the spectroscopic properties of the generated plasma were studied. The construction of the MPS was improved in two steps. Firstly, simulations, based on studies the electromagnetic field within the MPS, were used to predict optimal design modifications for the device’s interior. The simulations results were then validated experimentally by constructing an improved new version of the MPS.

To test the new MPS, the electrodynamic properties (electrodynamic characteristics and electric field distributions) of the new device and properties of microwave plasma generated in nitrogen, using optical emission spectroscopy (OES), were investigated. The OES method enabled records the emission spectrum of the plasma, allowing for the determination of the vibrational temperature *T*_vib_ and rotational temperature *T*_rot_ of the particles within the plasma^19–22^. The impact of conditions such as working gas flow rate and absorbed microwave power on the estimated values of *T*_vib_ and *T*_rot_ was tested.

## The waveguide-supplied coaxial-line-based nozzleless MPS

**Figure 2:**
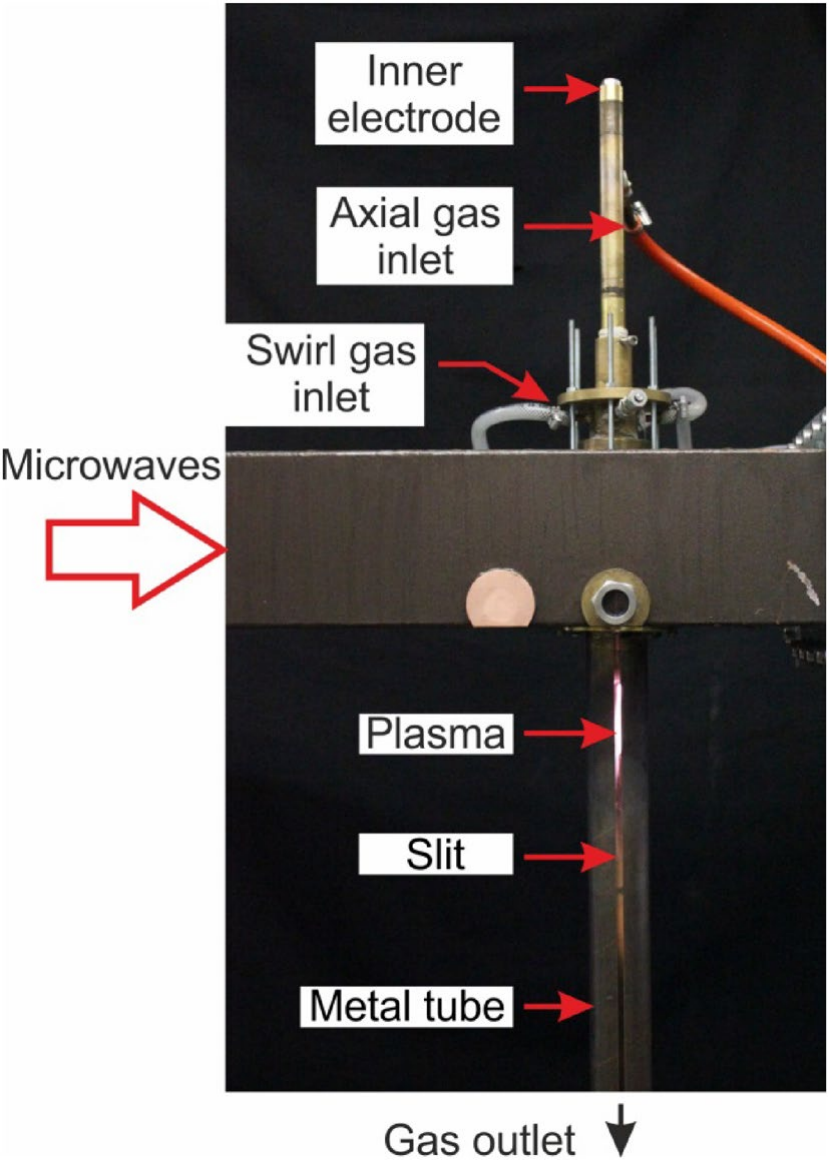
Photo of the waveguide-supplied coaxial-line-based nozzleless MPS.

The diagram of the MPS is presented in Fig. 1, while the photo is presented in Fig. 2. The MPS is based on a segment of a rectangular waveguide WR 975 with an inserted coaxial-line (inner electrode and metal tube). The waveguide of the MPS consists of two sections, each with a length of λ_g_/2, where λ_g_ = 437.7 mm is the microwave wavelength in the WR 975 waveguide. The first section, known as the tapered section (located between planes 1–1′ and 2–2′), linearly reduces the height of the WR 975 waveguide to the height of the second section. The second section, termed the reduced-height waveguide, is positioned between planes 2–2′ and 4–4′, featuring a reduced height of *b*_1_ = 31 mm compared to the standard WR 975 waveguide with a height of *b* = 123.9 mm. The reduced-height results in a higher electric field intensity in the area where the plasma is generated. The microwave power is delivered to the MPS through the rectangular waveguide at the input plane (plane 1–1′). At the output plane (plane 4–4) the movable plunger is attached, as shown in Fig. 1.

A special feature of this MPS design is the transition from a standard rectangular waveguide to the coaxial-line-based waveguide. This coaxial structure consisted of an inner electrode and a circular metal tube beneath the MPS waveguide. Microwave plasma in the form of a flame is generated inside a centrally positioned quartz tube within the reduced-height waveguide section (plane 3–3′). The inner and outer diameters of the quartz tube are 26 mm and 30 mm, respectively. The metal tube in the MPS serves as the outer conductor of the coaxial line, ensuring the integrity of the transmission line and completing the electrical circuit. Furthermore, the metal tube provides effective electromagnetic shielding, assists in heat dissipation, and offers mechanical protection for the inner components. The tube contains a vertical narrow slit that enables observation of the generated plasma. The plasma is generated in a gas (known as shielding gas) that is introduced into the device’s interior through four perpendicular inlets to the quartz tube that are also tangent to its inner wall. This method of introduction creates a swirling flow of the shielding gas inside the quartz tube, which protects the quartz wall from overheating (damage) and stabilizes the discharge^18^. The working gas, which is subjected to the conversion process, is introduced axially into the generated plasma through the inner electrode.

## The experimental setup

The experimental setup comprises the following components: a microwave source, a directional coupler, a waveguide transmission line for microwave propagation, the MPS, the movable plunger and a gas delivery system. The microwave source used in the setup is a magnetron microwave generator manufactured by Muegge, model MG 020KE-510KE. This source allows for the generation of microwaves at the frequency 915 MHz within a power range of up to 20 kW. The waveguide transmission line is constructed using standard WR 975 waveguide components. A directional coupler (with a coupling of 70 dB) connected to a digital dual-channel microwave power meter is positioned after the microwave source in the waveguide transmission line. This configuration enables real-time measurements of both the incident *P*_I_ and reflected *P*_R_ microwave power at the input plane of the MPS. The gas is delivered to the MPS at atmospheric pressure and its volumetric flow rate is regulated by a Mass Flow Controller regulator type. The details of the experimental setup can be found in the reference^18^.

## The measured electrodynamic characteristic of the MPS

**Figure 3:**
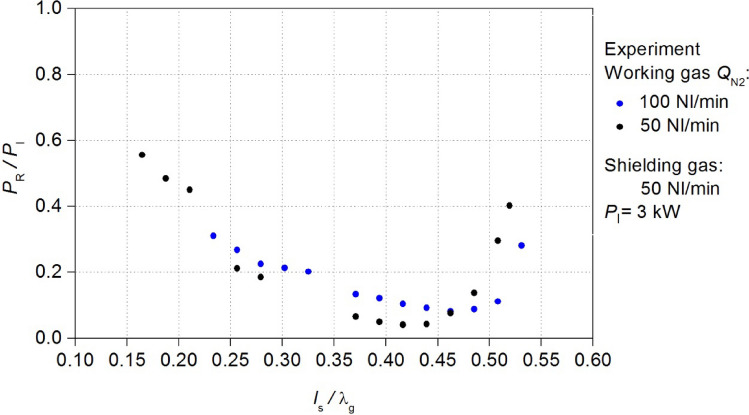
Measured electrodynamic characteristics of the MPS. The position of the movable plunger *l*_S_ is normalized to the wavelength λ_g_.

The electrodynamic characteristics of the MPS were measured for the microwave plasma in nitrogen as both working and shielding gas. The flow rate of the working gas *Q*_N2_ was 50 Nl/min and 100 Nl/min, while the shielding gas flow rate was 50 Nl/min. Under these conditions, the electrodynamic characteristics of the MPS were measured for the microwave power *P*_I_ = 3 kW (Fig. 3).

The measured electrodynamic characteristics have revealed that an increase in the working gas flow rate leads to a reduction in the energy efficiency of the MPS. For the flow rate *Q*_N2_ = 50 Nl/min, the minimum measured *P*_R_/*P*_I_ ratio was 0.04, whereas for *Q*_N2_ = 100 Nl/min, the *P*_R_/*P*_I_ ratio was 0.08, this indicates that the absorption of microwave power decreases as the working gas flow rate increases. Furthermore, the increase in the working gas flow rate narrowed the range *l*_S_/λ_g_ of plasma generation from < 0.16–0.52 > to < 0.23–0.53 >. Given the potential applications of the MPS in industries where the device is intended for high-flow gas conversion (several hundred litters per minute), the above observations reveal an especially unfavourable phenomenon. Namely, the decrease of the efficiency of the microwave power absorption *P*_A_ by the generated plasma with an increase of the working gas flow rate. The experiment has shown that the MPS needs to improve its energy efficiency, which was achieved in this work.

## Improving the construction of the MPS

The aim of this improvement was to increase the energy efficiency of the MPS. To achieve this goal, the following steps were taken: adopting a plasma model, using the adopted plasma model to simulate the optimal design of the MPS, building a new MPS based on these calculations, and validating it.

The purpose of calculating the optimal design of the MPS is to minimize the cost of improving the construction of the MPS. Although experimental improvement is possible, it involves expenses due to physical modifications to the interior of the MPS and a series of measurements of electrodynamic characteristics. Simulations can help to avoid costly processes and save time by providing predictions for improving the energy efficiency of a device.

### Assumed plasma model

This chapter provides a description of the plasma model and the method used to calculate the electrodynamic characteristics of the MPS. This work adopts the approach proposed by Nowakowska et al.^23,24^ for calculating the electrodynamic characteristics of the MPS. The crucial points of this method include defining the shape of the generated plasma, the distribution of electron density *n*_e_ and the electric permeability ε_p_ of the plasma.

**Figure 4:**
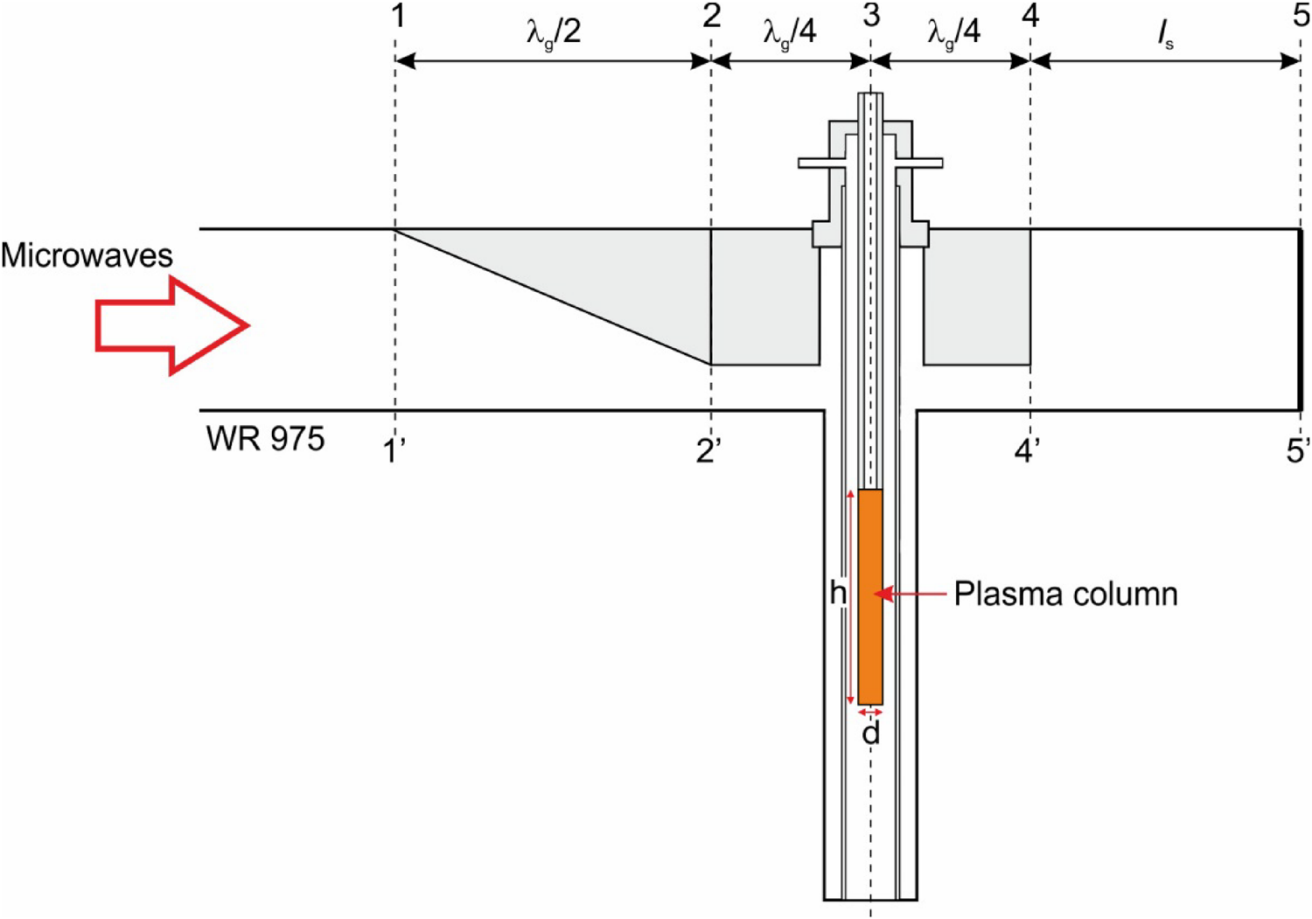
The assumed shape of the plasma column in the MPS.

Based on the experimental observations, it is assumed that the plasma takes the form of a cylindrical column with a diameter *d* and a length *h*. The axis of the plasma cylinder coincides with the axis of the coaxial line (plane 3–3), as shown in Fig. 4. For a flow rate of working gas *Q*_N2_ = 50 Nl/min, the values of *d* and *h* are 20 mm and 160 mm, respectively. For a higher flow rate, *Q*_N2_ = 100 Nl/min, the diameter *d* remains at 20 mm, while the height *h* is 140 mm. In both cases, it is assumed that the base of the plasma column is located just below the end of the inner electrode.

It is further assumed that the plasma is homogeneous, i.e., the electron density *n*_e_ in every point of the cylinder is the same. Besides, the relative electric permeability *ε*_p_ of the plasma is described using the Lorentz equation^23,24^:

1 $$\varepsilon_{p} = 1 - n/(1 - {\text{j}}s).$$

In Eq. (1), *n* = *n*_e_/*n*_c_ is the normalized electron density *n*_e_ relative to the critical electron density *n*_c_, *s* = ν/ω is the normalized electron collision frequency ν relative to the angular frequency ω = 2π*f*, and a j = (− 1)^1/2^. The *n*_c_ in the plasma is described by the equation^23,24^:

2 $$n_{{\text{c}}} = \, \omega^{{2}} \varepsilon_{0} m_{{\text{e}}} /e^{{2}} = { 1}.0{4 } \times {1}0^{{6}} {\text{m}}^{{ - {3}}} ,$$

where ε_0_ is the electric permeability of vacuum (ε_0_ = 8.85·10^–12^ F/m), *m*_e_ is the mass of an electron (*m*_e_ = 9.1·10^–31^ kg), and *e* is the charge of an electron (*e* = 1.6·10^–19^ C).

The RF module of the COMSOL Multiphysics software^25^ allows for the determination of the reflection coefficient of the microwaves Γ_in_ in the input plane of the MPS (plane 1–1′, Fig. 1) for any position *l*_S_ of the movable plunger. The value of the *P*_R_/*P*_I_ ratio is related to the coefficient Γ_in_ by the following equation:

3 $$\frac{{P_{{\text{R}}} }}{{P_{{\text{I}}} }}\left( {\frac{{l_{{\text{s}}} }}{{\lambda_{{\text{g}}} }}} \right) = \left| {\Gamma_{{{\text{in}}}} \left( {\frac{{l_{{\text{s}}} }}{{\lambda_{{\text{g}}} }}} \right)} \right|^{2} .$$

**Figure 5:**
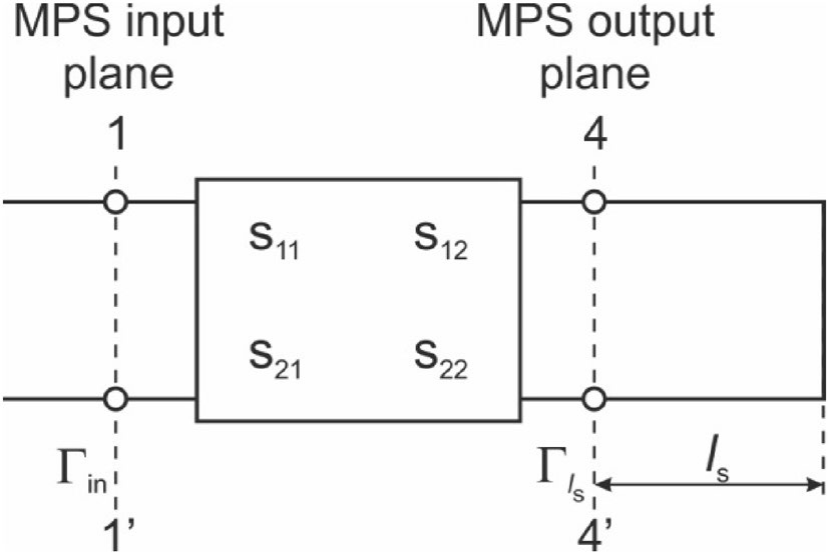
The MPS as the two-port network terminated with the short-circuited transmission line.

To determine the electrodynamic characteristics of the MPS, relations *P*_R_/*P*_I_ (*l*_S_/λ_g_), calculations must be performed for the full range of the normalized plunger positions (*l*_S_/λ_g_) which are time-consuming. Nowakowska et al.^23,24^ proposed a method to streamline this procedure. They assume that the MPS is the two-port network terminated with a section of a short-circuited transmission line of length *l*_S_ (Fig. 5).

Using the scattering matrix ***S*** of the two-port network, the coefficient Γ_in_ in the input plane, is described by the following equation^23,24^:

4 $$\Gamma_{{{\text{in}}}} (l_{{\text{s}}} ) = s_{11} + \frac{{s_{12} s_{21} \Gamma_{{l_{{\text{s}}} }} }}{{1 - s_{22} \Gamma_{{l_{{\text{s}}} }} }}.$$

In the Eq. (4), *s*_11_, *s*_12_, *s*_21_, and *s*_22_ are the elements of the ***S*** matrix, and Γ_ls_ represents the reflection coefficient of the microwaves from a terminated transmission line in the output plane of the two-port network, as illustrated in Fig. 5. The coefficient Γ_ls_ is given by^23,24^:

5 $$\Gamma_{{l_{s} }} = \frac{{z_{l} - 1}}{{z_{l} + 1}},$$

where *z*_l_ = jtg(2π*l*_s_/λ_g_) is impedance of the short-circuited transmission line of length *l*_S_, normalized to the characteristic impedance of the transmission line *Z*_l_.

To numerically determine the elements of the ***S*** matrix, simulations of the distribution of the electromagnetic field within the empty MPS connected to a matched load has to be conducted. For this purpose, The RF module of the COMSOL Multiphysics software^25^ was applied.

**Figure 6:**
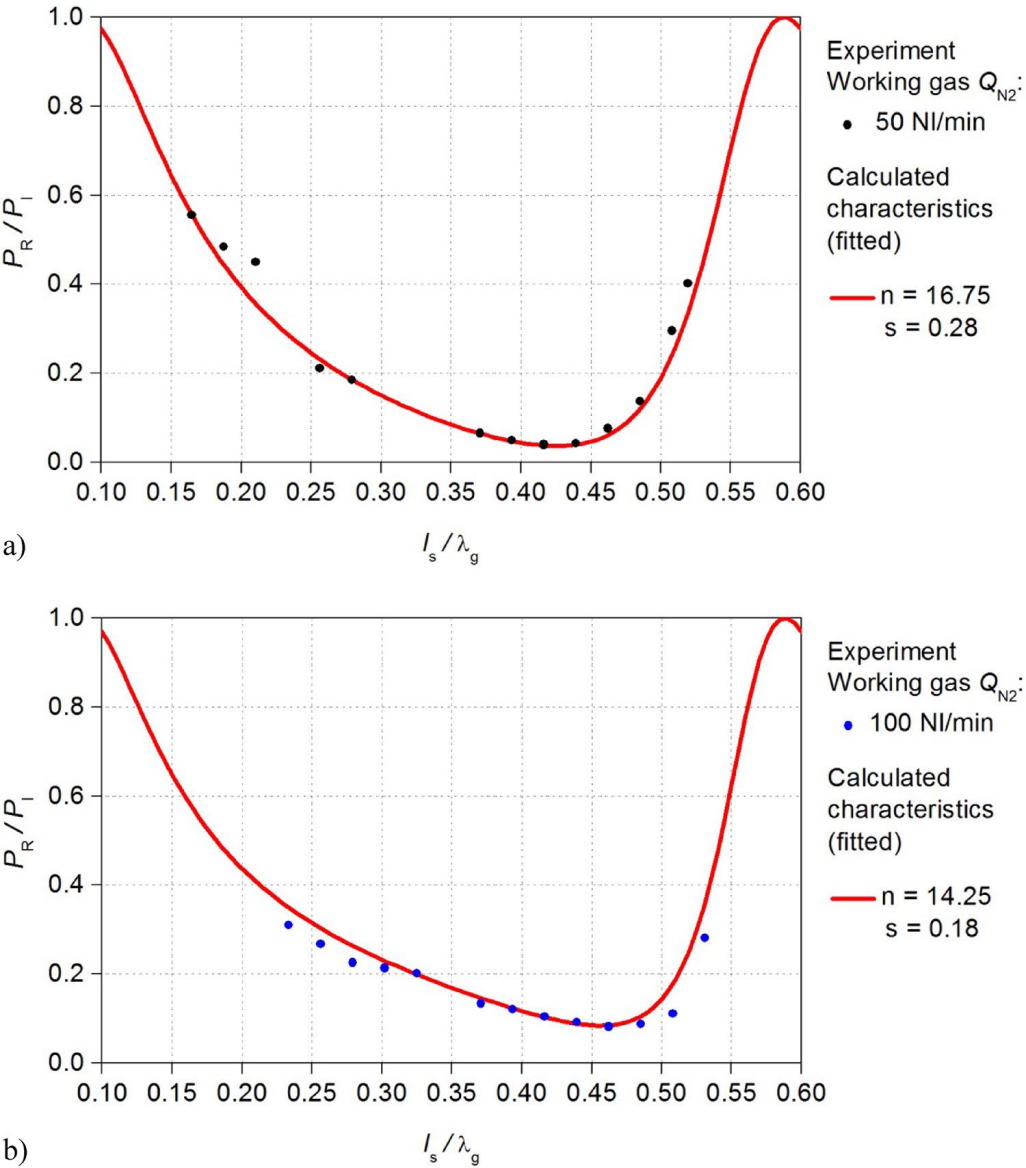
Comparison of the electrodynamic characteristics of the MPS for the flow rate of the working gas *Q*_N2_ (**a**) 50 Nl/min, (**b**) 100 Nl/min: experimental point and calculated curve.

In the methodology proposed by Nowakowska^23,24^, the fundamental factors shaping the *P*_R_/*P*_I_ (*l*_S_/λ_g_) relations are the parameters *n* and *s*, initially unknown. Experimentally, *n* and *s* can be determined, for instance, using optical emission spectroscopy (OES) techniques. In situations where experimental constraints preclude such determinations, numerical techniques can be employed to fit the calculated *P*_R_/*P*_I_ (*l*_S_/λ_g_) relations to the measured electrodynamic characteristics^26^. Aligning the calculated with the measured electrodynamic characteristics of the MPS permitted the derivation of parameters *n* and *s* within the used microwave plasma model. In the present case this aligning was performed using the least squares method. In the case of microwave discharge featuring the flow rate of the working gas *Q*_N2_ = 50 Nl/min, the best aligning was achieved with values of *n* and *s* amounting to 16.75 and 0.28, respectively, as shown in Fig. 6a). In contrast, for the case of *Q*_N2_ = 100 Nl/min, a concordance was achieved with *n* = 14.25 and *s* = 0.18 (Fig. 6b).

Determining the parameters *n* and *s* enables the estimation of electron density *n*_e_ and collision frequency ν in the generated plasma, see Eq. (1). For *Q*_N2_ = 50 Nl/min, the estimated electron density was *n*_e_ = 1.74 × 10^11^ cm^−3^, with an electron collision frequency of ν = 1.6 × 10^9^ s^−1^, in the case of *Q*_N2_ equal to 100 Nl/min *n*_e_ = 1.48 × 10^11^ cm^−3^ and ν = 1 × 10^9^ s^−1^. It is important to note that the electron density values obtained in our studies are lower than those commonly reported in the literature. This discrepancy is primarily attributable to the assumption of a homogeneous plasma. Nevertheless, the model accurately predicts the trends in electron density variations in the plasma under different discharge conditions.

The increase in the gas flow rate impacts the length of the plasma flame, which in affects the impedance of the generated microwave discharge. Additionally, the applied plasma model and conducted simulations indicate that an increase in the flow rate leads to a reduction in electron concentration and collision frequency. The change in impedance and the decrease in electron concentration and collision frequency are the primary reasons for the observed reduction in the absorption of microwave power with higher gas flow rates.

### Optimizing the MPS design

**Figure 7:**
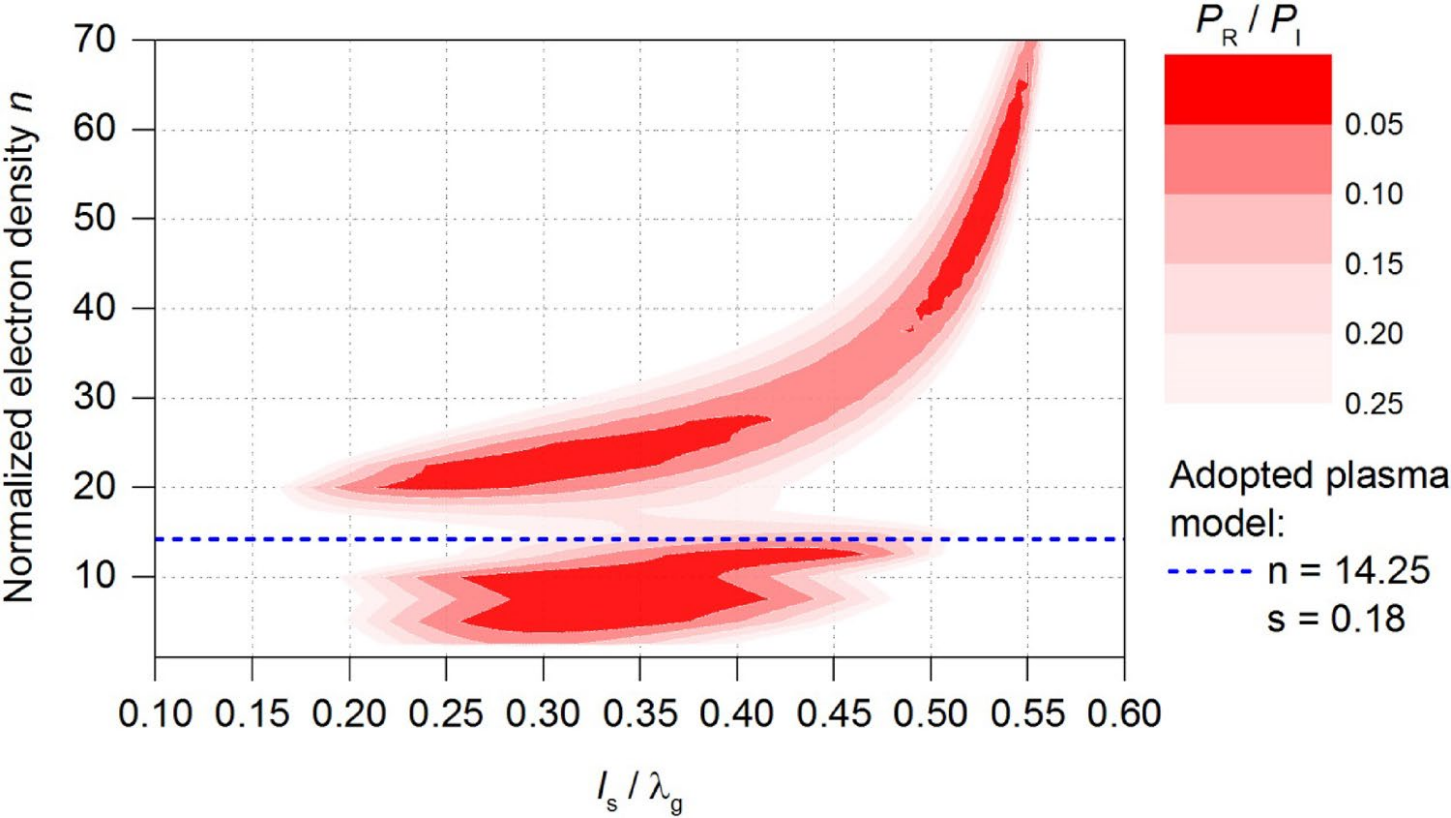
Map of the stable plasma generation area—starting point in improvement of the energy efficiency of the MPS. The dashed line marks the value of the normalized electron density *n* of the adopted plasma model.

The starting point in the improvement of energy efficiency of the MPS was the plasma model obtained for the case of the flow rate *Q*_N2_ = 100 Nl/min (*d* = 20 mm, *h* = 140 mm, *n* = 14.25, *s* = 0.18). For the adopted plasma model, Fig. 7 presents calculated electrodynamic characteristics of the MPS in a form of a two-dimensional contour map, known as *stable plasma generation area*^24,27^. This map illustrates the calculated *P*_R_/*P*_I_ ratio as a function of two variables: the normalized position of the movable plunger (*l*_S_/λ_g_) and the normalized electron density *n*. The electrodynamic characteristics ranges from 0 to 1. However, the focus of this work was on the cases in which the *P*_R_/*P*_I_ values lie within the range of 0 to 0.25, in order to deal only with the cases where the microwave power is most efficiently absorbed by the generated plasma.

**Figure 8:**
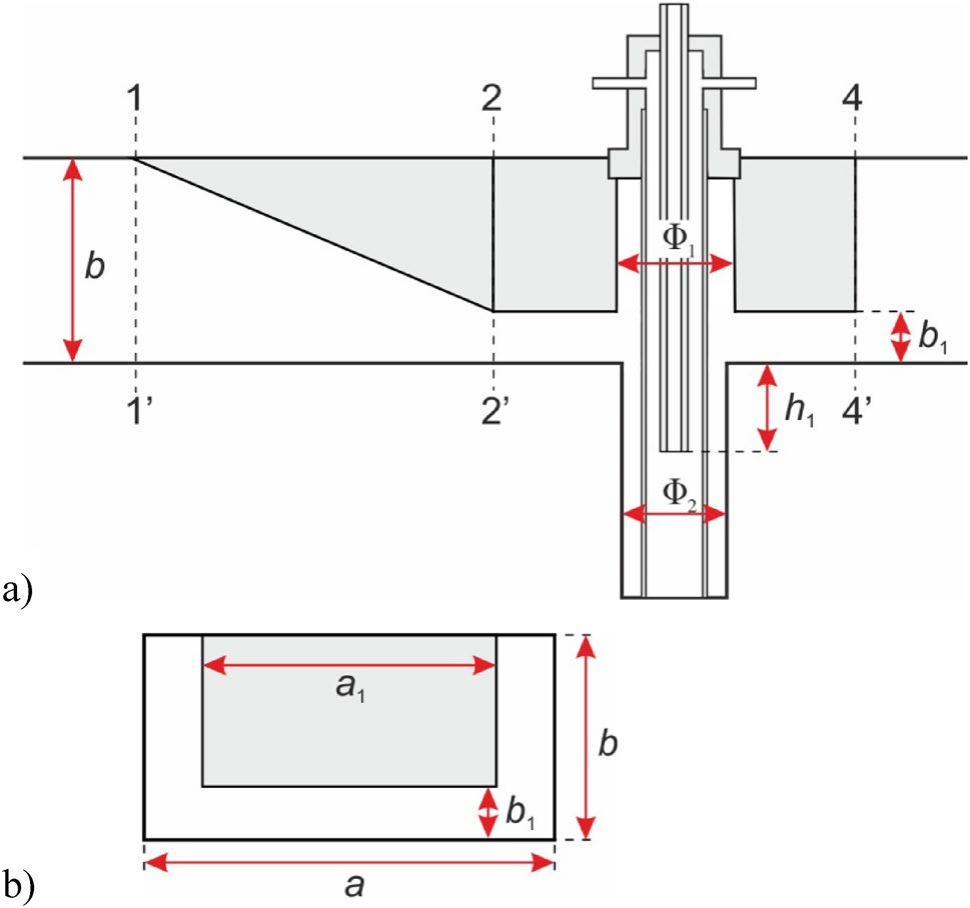
The analysed construction dimensions in the MPS: (**a**) longitudinal cross-section, (**b**) transverse cross-section.

The map shown in Fig. 7 enables the fast assessment of MPS energy efficiency. The assesses is based on the size of the marked red area (low *P*_R_/*P*_I_ values). The improvement of the MPS involves searching for such its inner dimensions that, on the plotted map (Fig. 7), will ensure the minimum *P*_R_/*P*_I_ ratio within close range of the normalized electron density *n* of the adopted plasma model. This will ensures efficient transfer of microwave energy in the considered case of microwave plasma. In practice, the improvement process involves creating the map for each change in the design of the MPS, and then comparing the maps obtained to analyse the area changes^24,27^. The best version of the MPS is determined by the largest areas marked red on the plotted map. This work analysed the influence of the following construction dimensions in the MPS on the map of the stable plasma generation area:

- *b*_1_—height of the reduced-height section,
- *h*_1_—distance from the end of the inner electrode to the reduced-height section,
- Φ_1_—inner diameter of the upper section of the coaxial line,
- Φ_2_—inner diameter of the lower section of the coaxial line,
- *a*_1_—width of the ridge,

The analysed dimensions (*b*_1_, *h*_1_, Φ_1_, Φ_2_,* a*_1_) are marked in Fig. 8. In the improvement, it was decided to replace the tapered and reduced-height section with a ridge of width *a*_1_.

**Figure 9:**
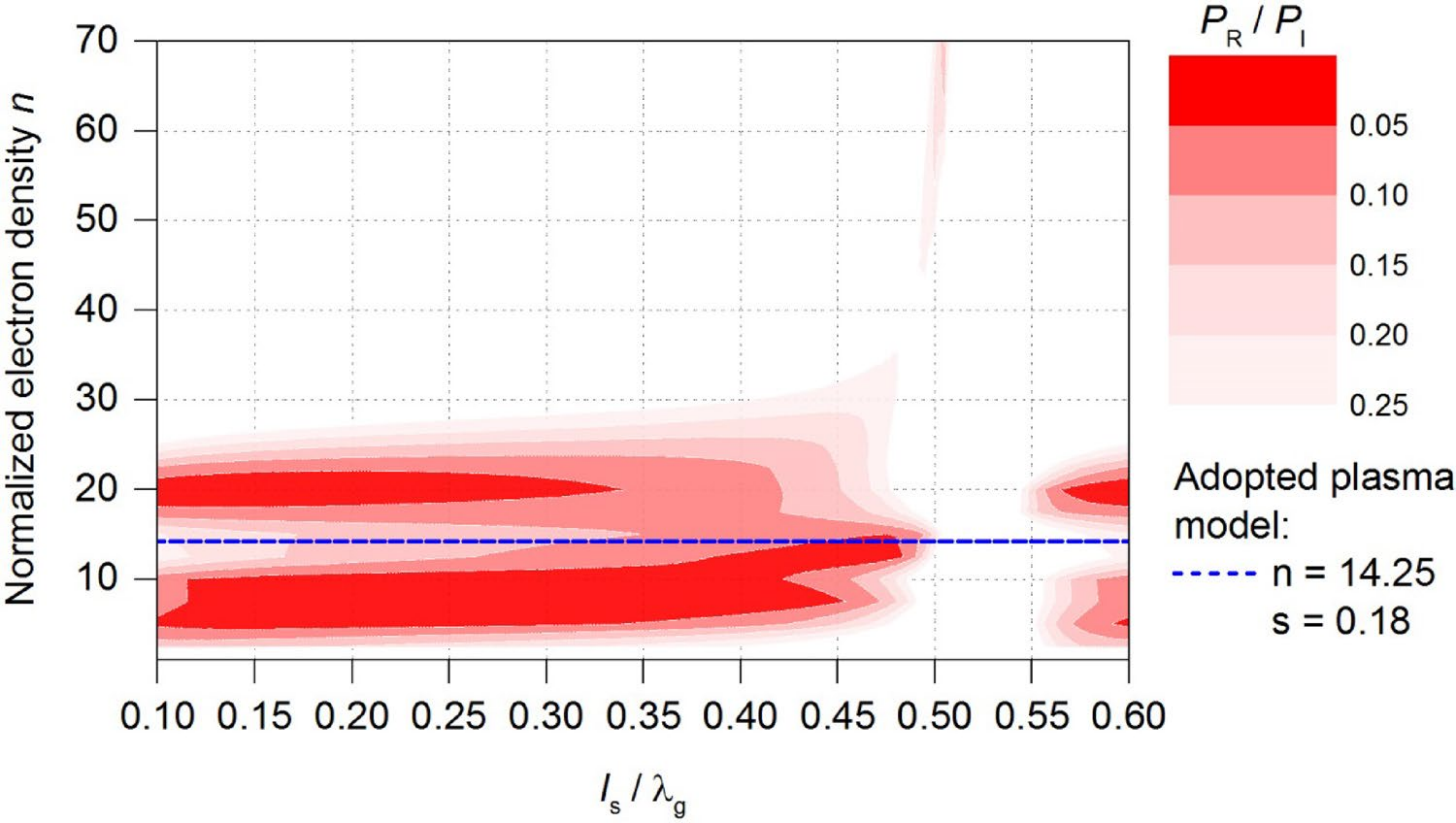
Map of the stable plasma generation area of the MPS after the improvement.

In improving energy efficiency in the device, a series of maps of the stable plasma generation area were plotted. However, in this work, the presentation of these results is limited to the map calculated for the MPS construction after the improvement, Fig. 9.

In comparison to Fig. 7, which served as the starting point, the improvement now ensures a low *P*_R_/*P*_I_ ratio in close to the *n* value of the adopted plasma model across a wide range of movable plunger positions, Fig. 9. This indicates a potential significant increase in the energy efficiency of the MPS. The new construction dimensions of the MPS are presented in Table 1, alongside with their values before improvement. The analysis indicated that the distance *h*_1_ should remain unchanged, while the inner diameter of both the upper and lower sections of the coaxial line should be equal Φ_1_ = Φ_2_. It is worth noting that the significant increase in the device’s energy efficiency is mainly due to the introduction of the ridge of width *a*_1_ into the interior of the MPS.

**Table 1:** The analysed construction dimensions in the MPS.

Before improvement	After improvement
*b*_1_ = 31 mm	*b*_1_ = 35 mm,
*h*_1_ = 40 mm	*h*_1_ = 40 mm
Φ_1_ = 53 mm	Φ_1_ = Φ_2_ = 52 mm
Φ_2_ = 48 mm	
*a*_1_ = *a* = 247*.*7 mm	*a*_1_ = 0, 7*a* = 173.4 mm

**Figure 10:**
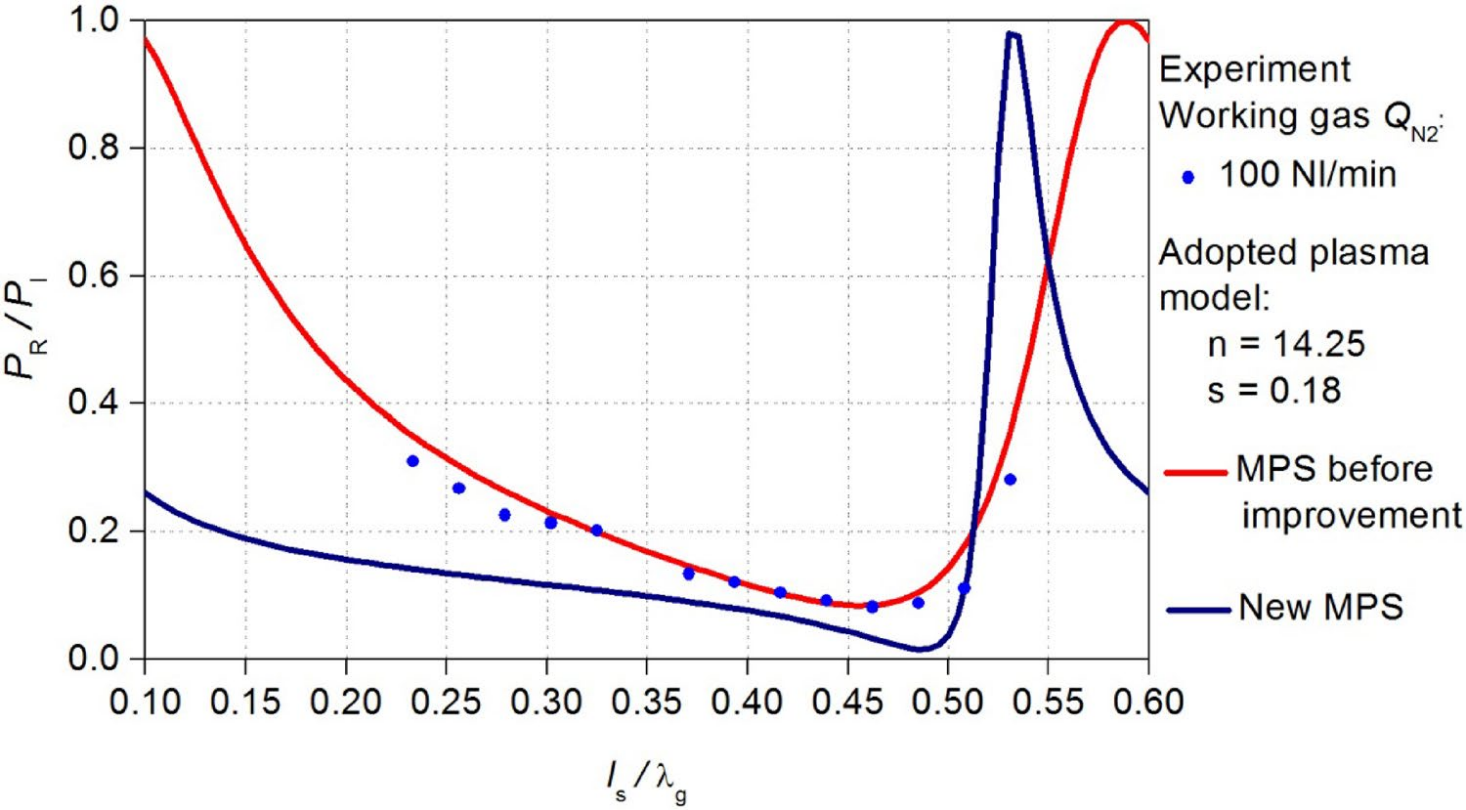
Comparison of the calculated electrodynamic characteristics of the new MPS with the characteristics before improvement, adopted plasma model *Q*_N2_ = 100 Nl/min.

**Figure 11:**
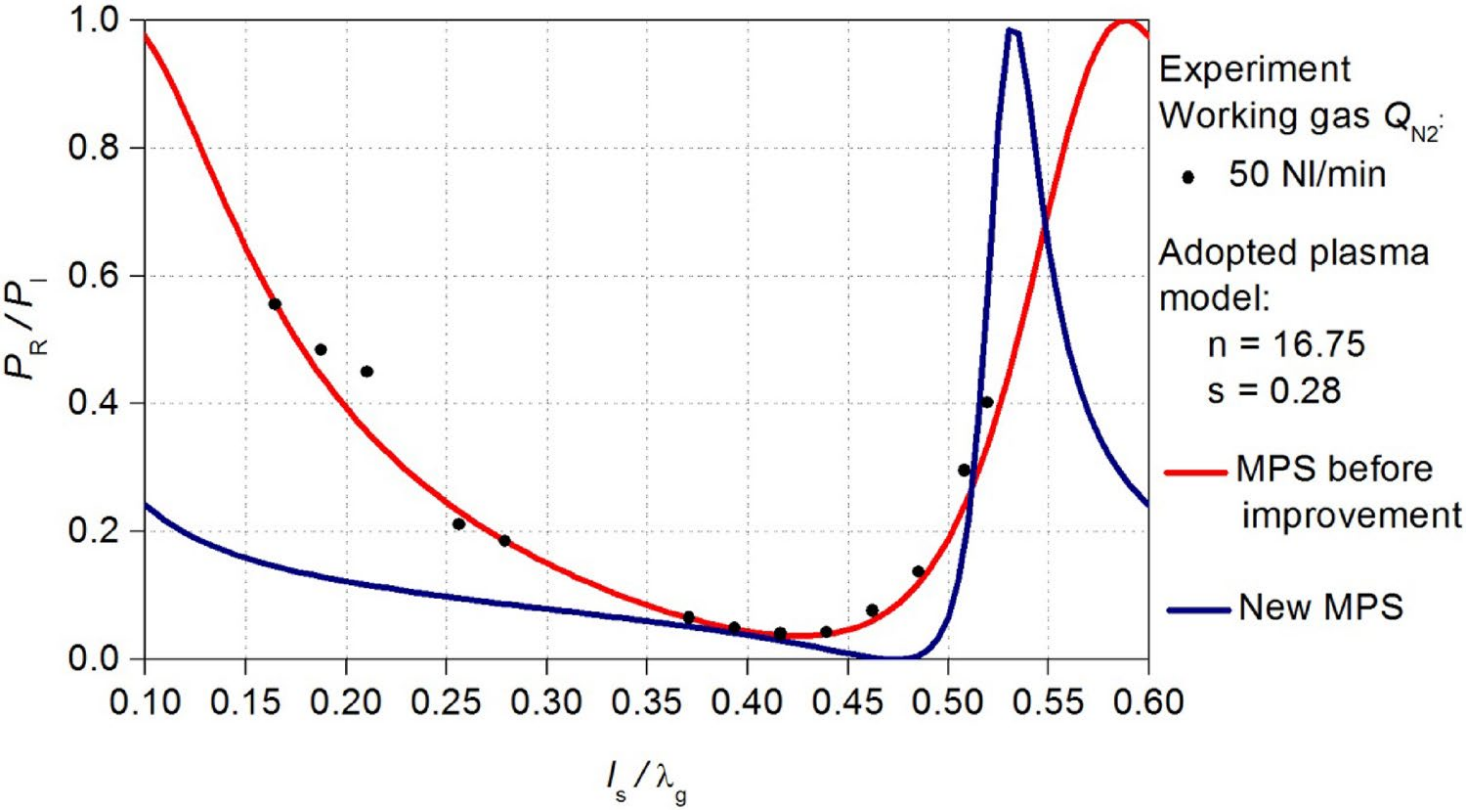
Comparison of the calculated electrodynamic characteristics of the new MPS with the characteristics before improvement, adopted plasma model *Q*_N2_ = 50 Nl/min.

The MPS, after improvement of the energy efficiency will be referred to as the new MPS. Using the new MPS design, new electrodynamic characteristics *P*_R_/*P*_I_ (*l*_s_/λ_g_) were calculated. In Figs. 10 and 11 the obtained relations are compared with the characteristics before the improvement. These news electrodynamic characteristics illustrate a notable increase in the energy efficiency of the new MPS. Now, within the *l*_s_/λg range of < 0.4–0.45 > the *P*_R_/*P*_I_ ratio approaches a value close to zero. This improvement is observed for both cases of considered working gas flow rate *Q*_N2_.

### The new MPS

**Figure 12:**
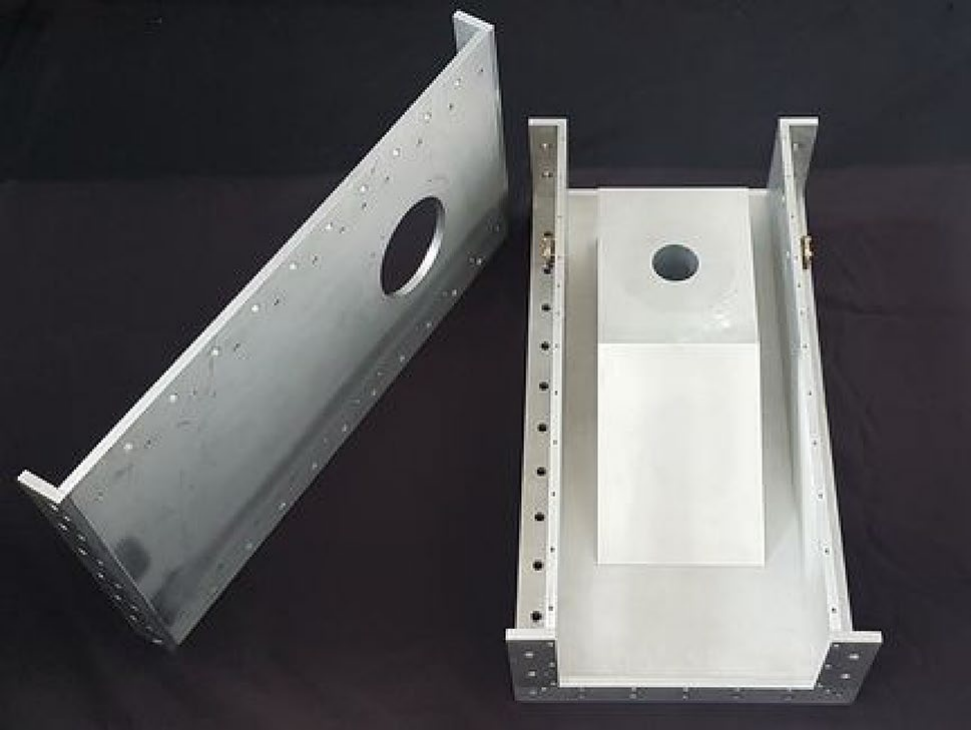
Novel modular waveguide construction of the new MPS.

To confirm the theoretical predictions, the new MPS based on the WR 975 waveguide standard and dimensions obtained from improvement has been manufactured. In the new MPS, an innovative approach to the construction of the waveguide was employed, namely, a modular construction. Each of the new MPS waveguide walls constitutes a separate component. This offers a broad range of possibilities for future device modifications tailored to specific applications. A photo of the new MPS modular waveguide construction is shown in Fig. 12.

The components forming the new MPS waveguide are made of aluminium plates, while the coaxial line and the inlet collector, along with their mountings, are brass. Constructing the largest MPS elements from aluminium will reduce the overall device weight. Meanwhile, using brass will ensure durability and heat resistance for other components. The tip of the inner electrode of the coaxial line was made of tungsten.

**Figure 13:**
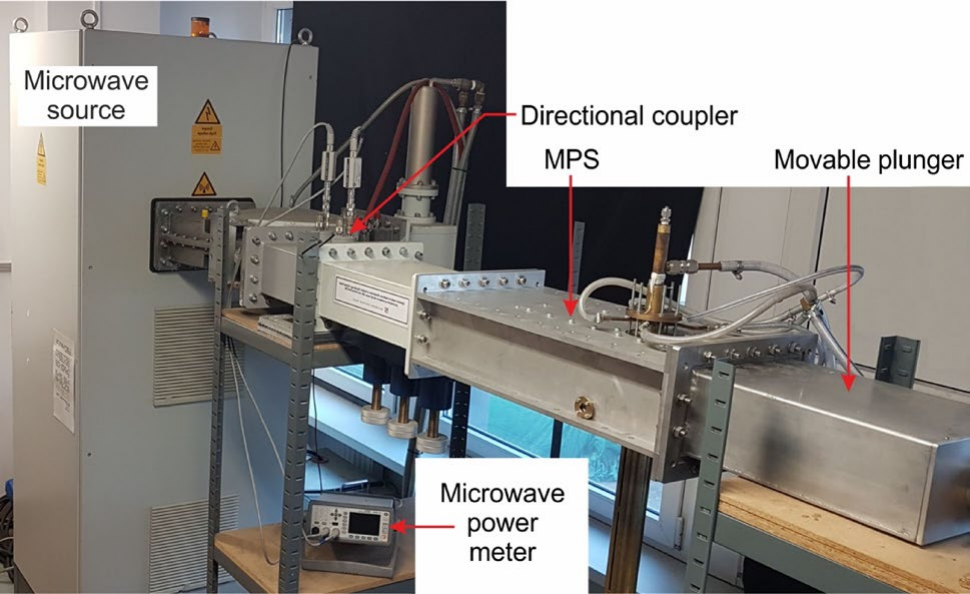
Photograph of the new MPS in the waveguide transmission line of the experimental setup.

**Figure 14:**
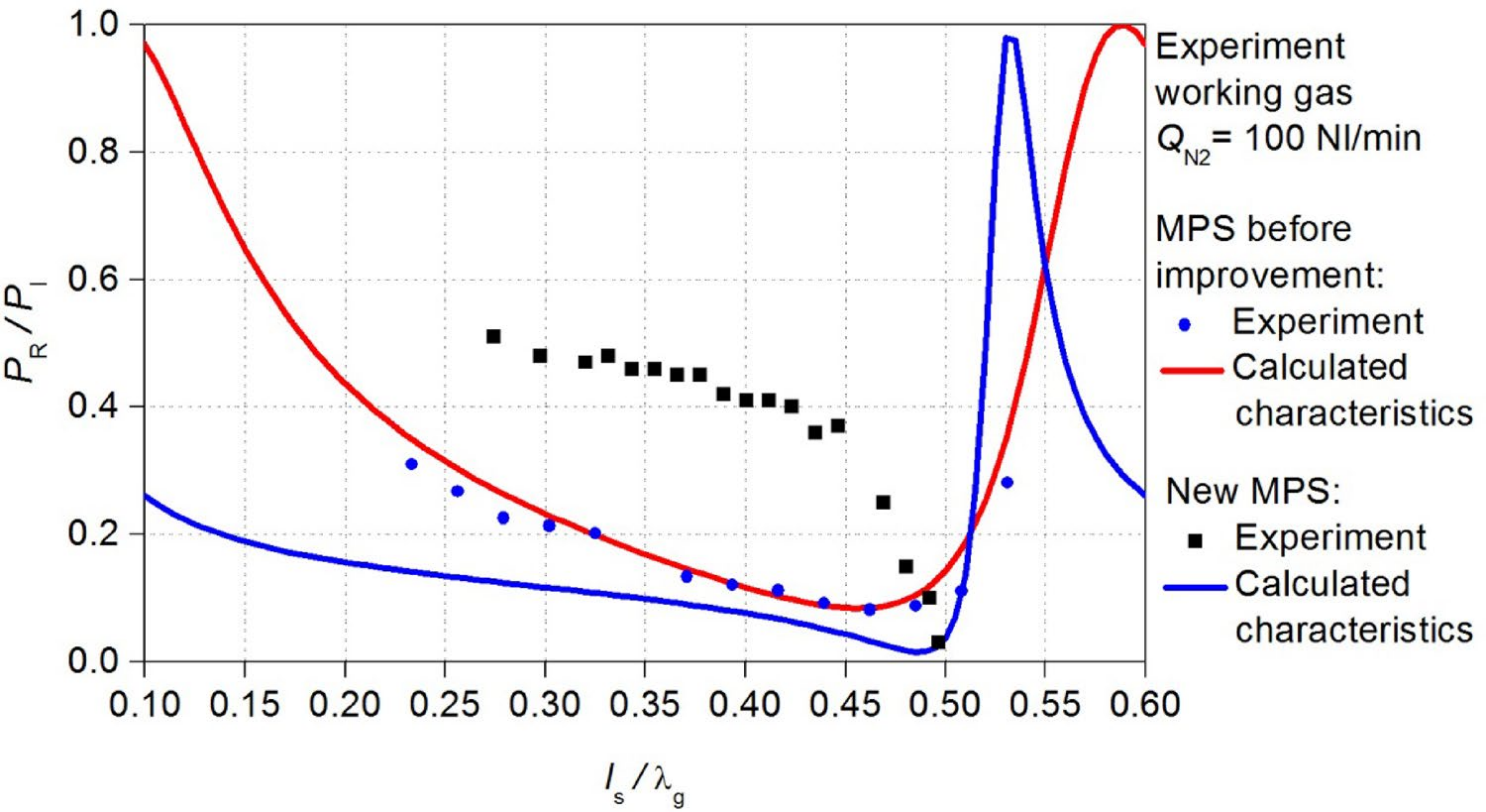
The measured electrodynamic characteristics (black squares) of the new MPS for *Q*_N2_ = 100 Nl/min. The new characteristics can be compered the previous results for the MPS before improvement.

**Figure 15:**
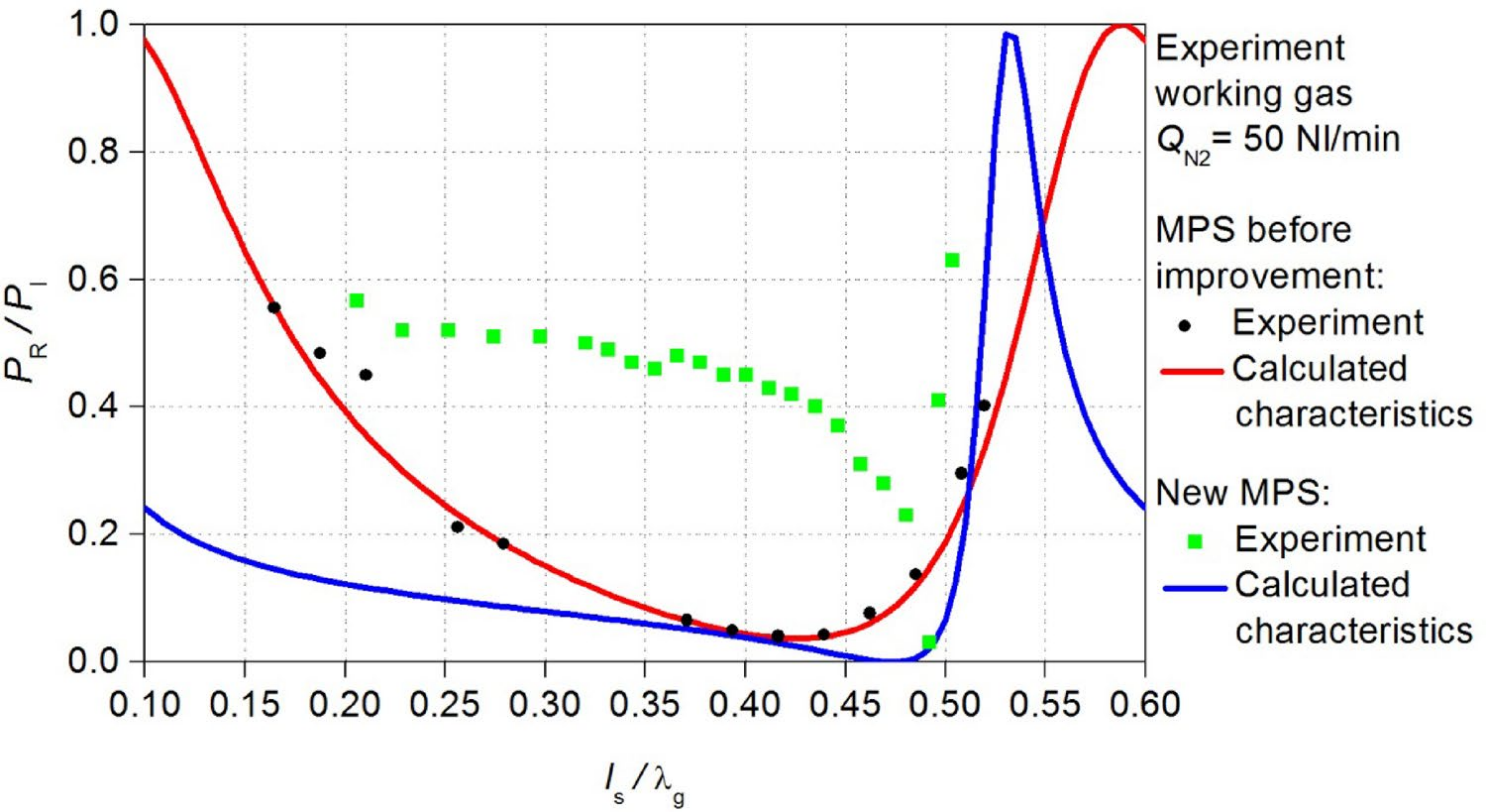
The measured electrodynamic characteristics (black squares) of the new MPS for *Q*_N2_ = 50 Nl/min. The new characteristics can be compered the previous results for the MPS before improvement.

The new MPS was placed in the waveguide transmission line of the experimental setup. A photograph of the new MPS is shown in Fig. 13. To validate the theoretical predictions, measurements of the electrodynamic characteristics of the new MPS were conducted under the same conditions as previously. The results are presented in Figs. 14 and 15. In these figures, the newly measured electrodynamic characteristics are compared with the previous results.

The validation indicated that the theoretical predictions align with measurements only in the vicinity of the position *l*_s_/λ_g_ = 0.5. Despite achieving a reduction in the reflected microwave power (the *P*_R_/*P*_I_ ratio lower than 3% at the position near *l*_s_/λ_g_ = 0.5 where the minimum characteristics occurs) this effect is not entirely satisfactory. The introduced modifications in the new MPS construction resulted in increase in the value of the reflected microwave power in the remaining range of the movable plunger position. Nevertheless, the range *l*_S_/λ_g_ of plasma generation in the new MPS and value of the *P*_R_/*P*_I_ ratio did not undergo significant changes, by doubling the volumetric flow rate of the working gas. This suggests the increased resilience of the device to the parameters of the generated microwave discharge operation.

The observed disparities between the electrodynamic characteristics of the new MPS, as determined through experimental measurements, and the theoretical predictions is probably result of the simplifications introduced in the used plasm model. Namely, due to the assumption of homogeneous plasma conditions with constant relative electric permeability. This approximation enabled a simplification of the calculations, leading to a notable reduction in the required computational time. It should also be noted that the movement of the movable plunger during the measurements of the electrodynamic characteristics alters an electromagnetic field distribution inside the MPS. This, in turn, influences the amount of microwave power absorbed by the generated plasma, leading to changes in electron density, dimensions of the discharge, etc. Additionally, focusing solely on nitrogen plasma may limit the applicability of the new MPS, as energy efficiency may not necessarily be the same for microwave discharges in other working gases, e.g. carbon dioxide, methane. However, the employed modular construction of the plasma source enables allows for successive improvements and modifications, such as adapting the device for hydrogen production from liquid or gaseous hydrogen carriers. The experience gained during this investigation will allow the construction of more sophisticated and efficient devices in the future.

## Investigations on microwave discharge in nitrogen in the new MPS

**Figure 16:**
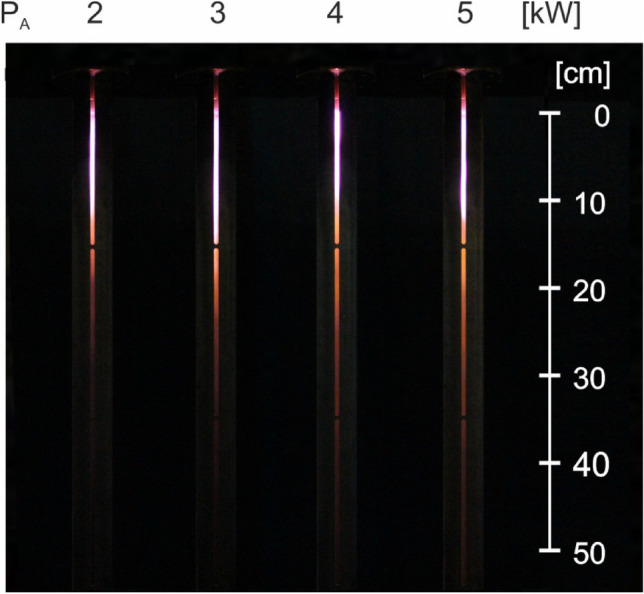
Photos of microwave plasma generated in nitrogen for different values of absorbed microwave power *P*_A_, *Q*_N2_ = 100 Nl/min. View through the slit in the metal tube of the coaxial-line-based waveguide.

**Figure 17:**
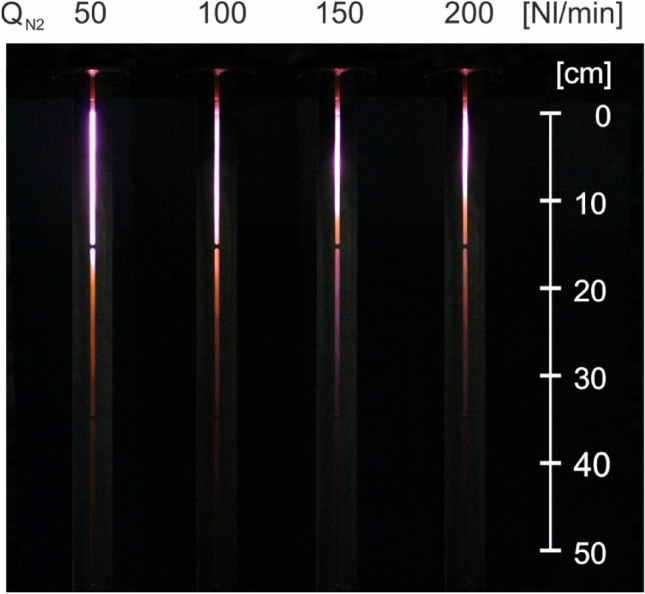
Photos of microwave plasma generated in nitrogen for different values of working gas flow rate *Q*_N2_, *P*_A_ = 3 kW. View through the slit in the metal tube of the coaxial-line-based waveguide.

Figures 16 and 17 present photos of the plasma flames to illustrate how the length of the flame changes with absorbed microwave power and working gas flow rate. The figures show that the length of the plasma flame decreases as the working gas flow rate increases and increases with the absorbed microwave power. In the tested range of absorbed microwave power (2–5 kW) and working gas flow rate (50–200 Nl/min), the length of the plasma flame varied between 8 and 23 cm. The generated plasma has a cylindrical shape with a base diameter approximately equal to the outer diameter of the inner electrode.

### Optical emission spectroscopy of microwave plasma generated in nitrogen

**Figure 18:**
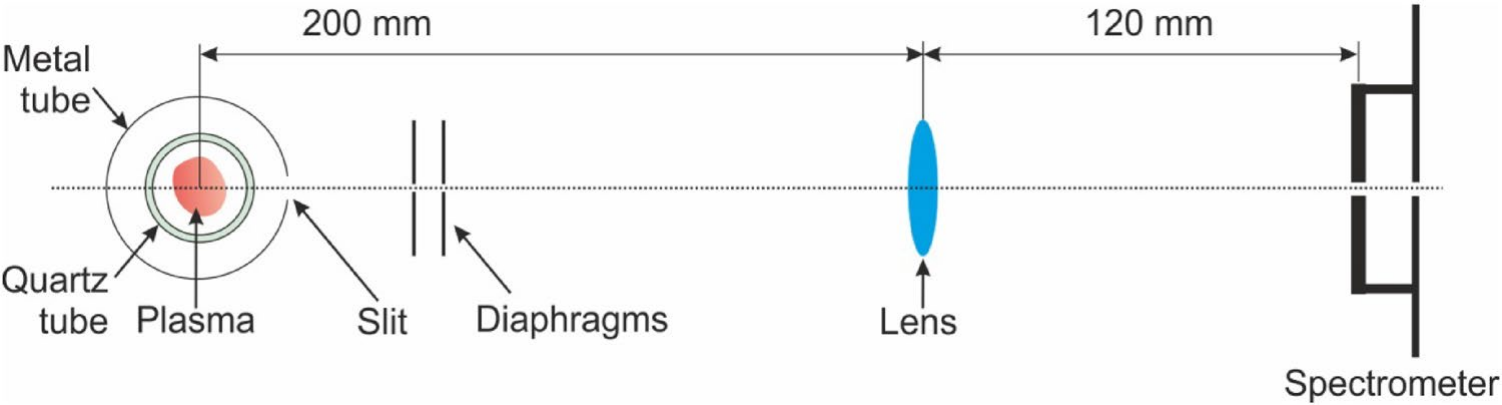
Diagram of the optical setup. Top view.

In OES investigations a simple optical setup with diagrams and lens, as illustrated in Fig. 18, was used. The radiation from the plasma was directed through a pair of diaphragms, each with a 1 mm pinhole, and a quartz lens onto a slit at the entrance of the spectrometer. The diaphragms ensured that only an almost parallel beam of radiation from the selected part of the generated plasma entered the spectrometer. The lens, which had a focal length of 75 mm, was positioned 200 mm from the centre of the microwave plasma and 120 mm from the spectrometer, perpendicular to the waveguide transmission line. The microwave discharge spectrum was recorded using a DK-480 spectrometer (CVI) equipped with diffraction gratings of 1200 and 3600 lines per mm and a CCD camera SBIG model ST-6, with a 242 by 750 pixel matrix.

The measurement area for recording the emission spectra of the microwave plasma, approximately 1.5 mm^2^, was established using geometric optics principles. Since the emission area is small and within this area, the plasma appears to be relatively homogeneous, spatial resolution was not introduced for this measurement.

**Figure 19:**
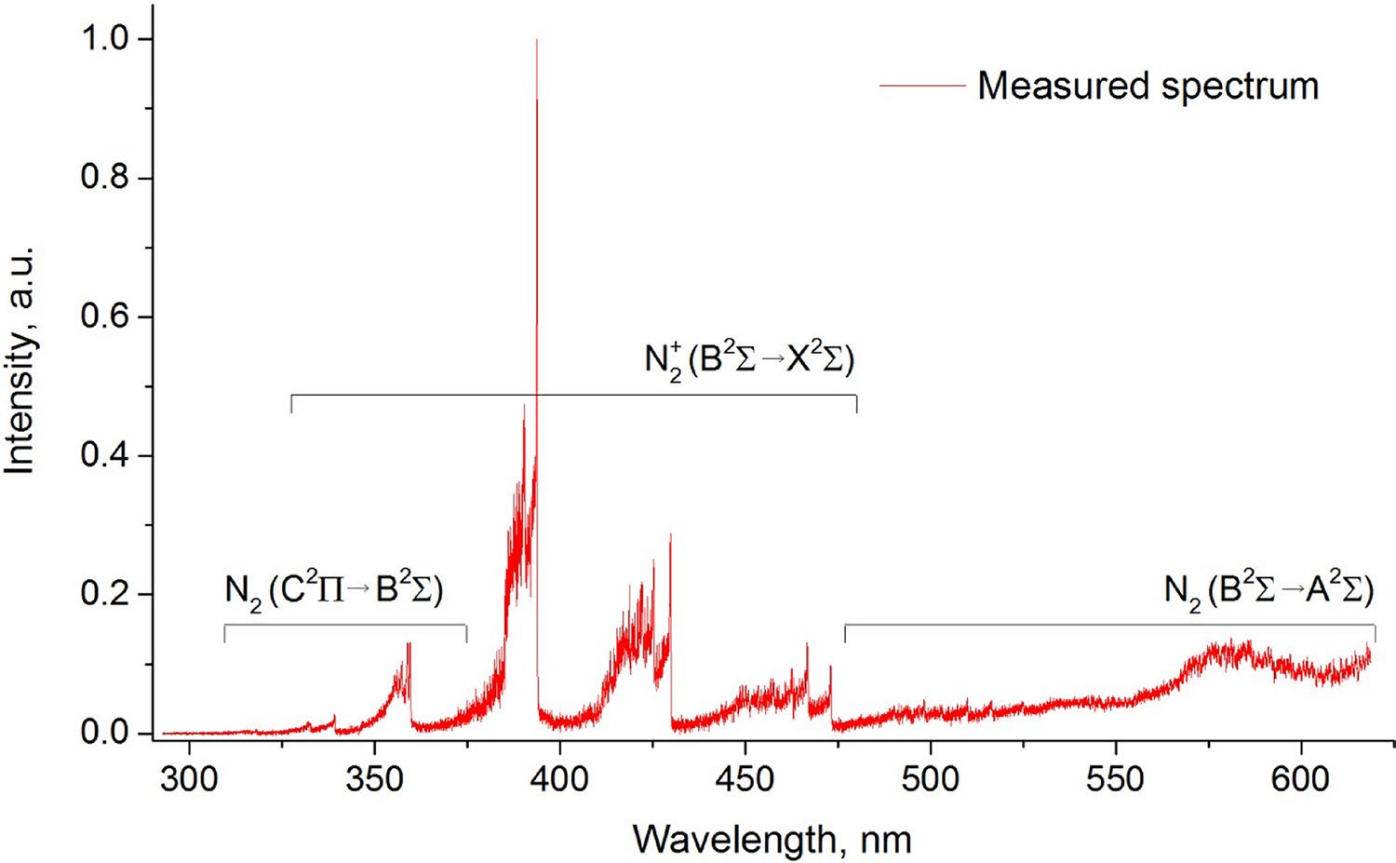
Spectrum of the microwave plasma in nitrogen (*P*_A_ = 3 kW, *Q*_N2_ = 50 Nl/min) measured 10 mm below the inner electrode.

Figure 19 shows an example of the measured spectrum of the microwave plasma in the wavelength range of 300–600 nm for the power *P*_A_ = 3 kW is presented. In this figure, three partially overlapping bands of nitrogen molecule and nitrogen ion are visible. The first dominant band corresponds to the nitrogen ion molecule:

- N_2_^+^ (1−) B^2^Σ → X^2^Σ, *the first negative system*,

and two weaker bands correspond to the nitrogen molecule:

- N_2_ (2 +) C^3^Π → B^3^Π, *the second positive system*,
- N_2_ (1 +) B^3^Π → A^3^Σ, *the first positive system*.

To determine the vibrational and rotational temperatures, the intensity of entire molecular bands must be measured^28^. However, this involves measuring a wide spectral range, which results in lower resolution of the measured spectrum. The method for determining the values of the *T*_vib_ and *T*_rot_ from the measured low-resolution spectrum involves fitting it to a simulated spectrum^21,22^.

Analyzing low-resolution spectra can be challenging due to overlapping bands when recording the emission from various molecules. In such situation, to determine the correct values of the *T*_vib_ and *T*_rot_, it is best to select a spectral range where different bands do not overlap or where one band clearly dominates over the others. In this work, the following spectral ranges have been selected for the determination of the vibrational and rotational temperatures of nitrogen molecules and nitrogen molecular ions:

- 306–318 nm—system N_2_ (2 +),
- 380–392 nm—system N_2_^+^ (1 −).

**Figure 20:**
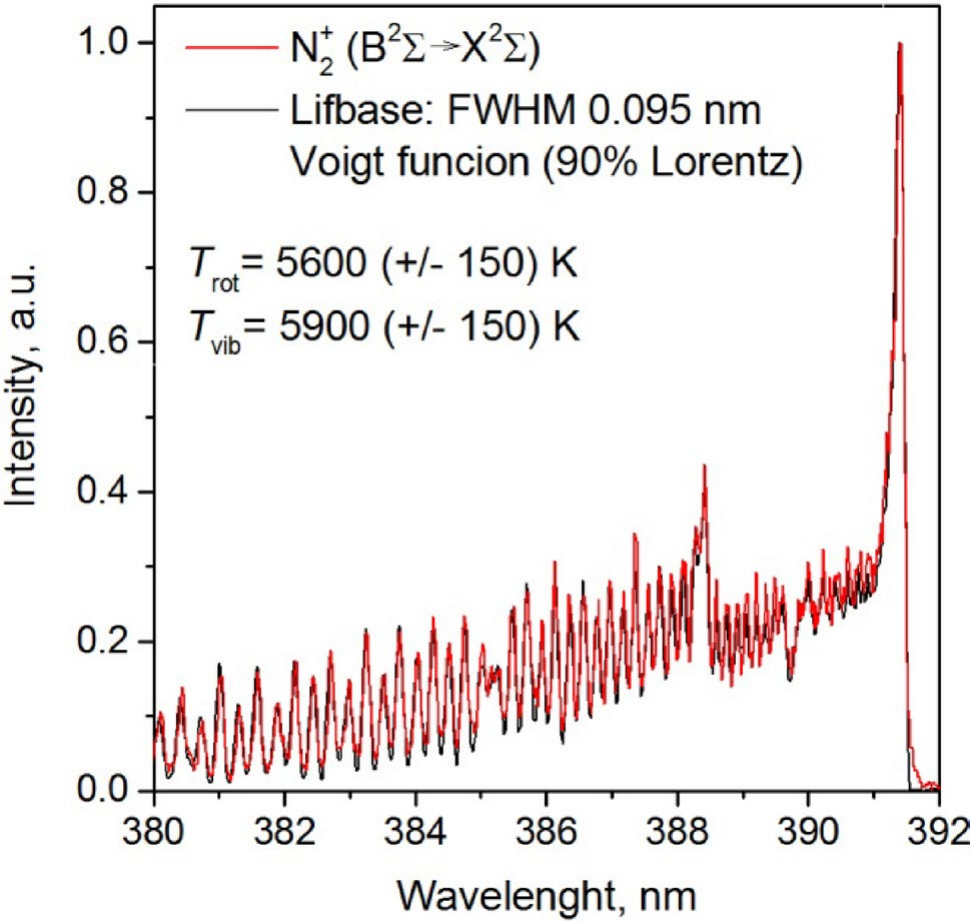
Comparison of the measured spectrum with the simulated spectrum obtained in the Lifbase.

The Lifbase^29^ software was used to generate the simulated spectra. An example of the comparison of the measured spectrum with the simulated spectrum is shown in Fig. 20. The Figure presents an example of the emission spectrum of the N_2_^+^ molecule ion in the range of 380–392 nm, which was separated from the background and normalized. The selected spectrum fragment has been compared with the spectrum obtained in the Lifbase program. The program simulations were conducted using a Voigt function (90% of the Lorentzian profile). The full width at half maximum (FWHM) of the Voigt function was set to 0.095 nm. The agreement between the measured and simulated spectra was determined using the least squares method. The average measurement error was approximately 200 K. The best agreement was achieved for the vibrational temperature *T*_vib_ of 5900 K and the rotational temperature *T*_rot_ of 5600 K.

**Figure 21:**
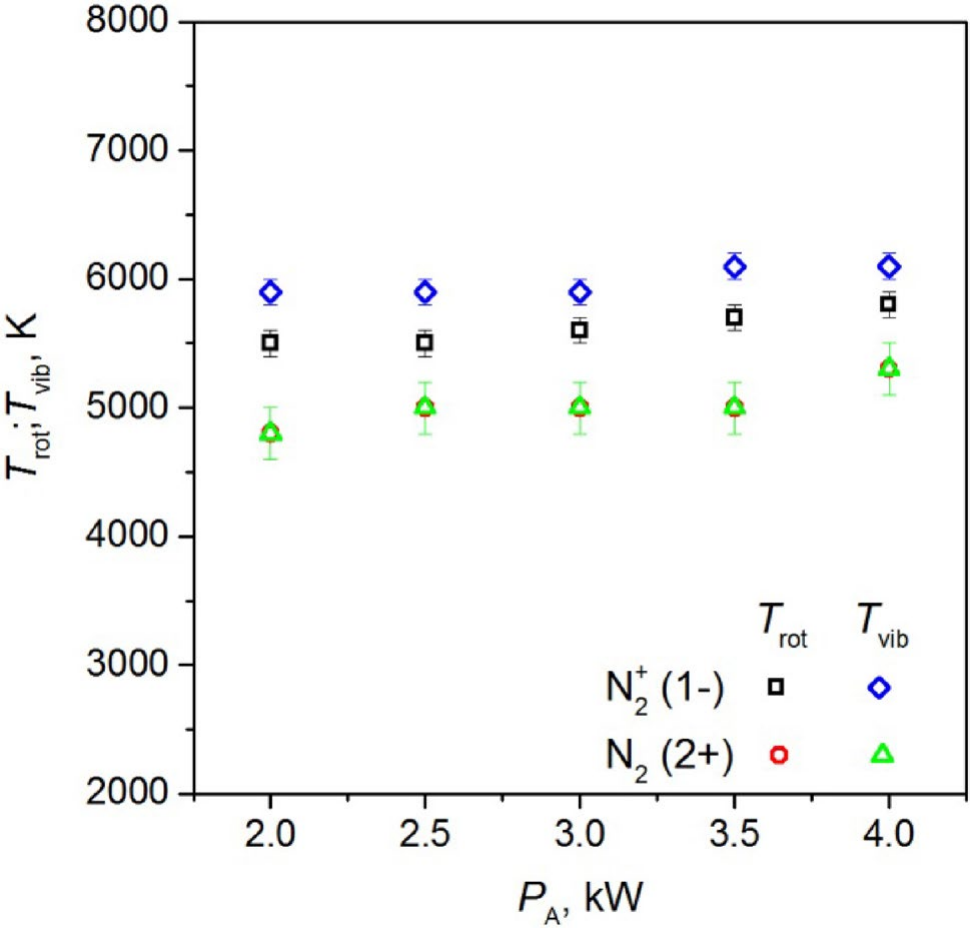
The measured values of the *T*_vib_ and *T*_rot_ for nitrogen molecules and nitrogen molecule ions as a function of absorbed microwave power *P*_A_, *Q*_N2_ = 50 Nl/min. Measured 10 mm below end of the inner electrode.

**Figure 22:**
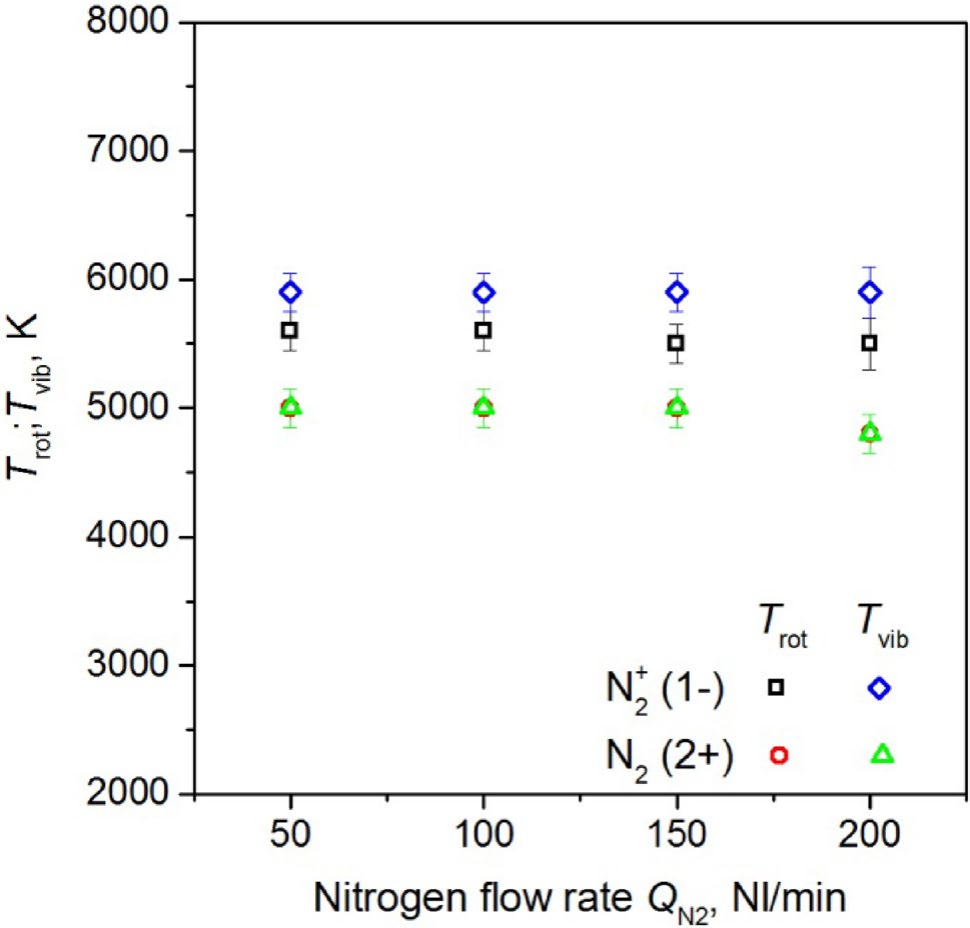
The measured values of the *T*_vib_ and *T*_rot_ for nitrogen molecules and nitrogen molecule ions as a function of volumetric flow rate of nitrogen *Q*_N2,_ *P*_A_ = 3 kW. Measured 10 mm below end of the inner electrode.

**Figure 23:**
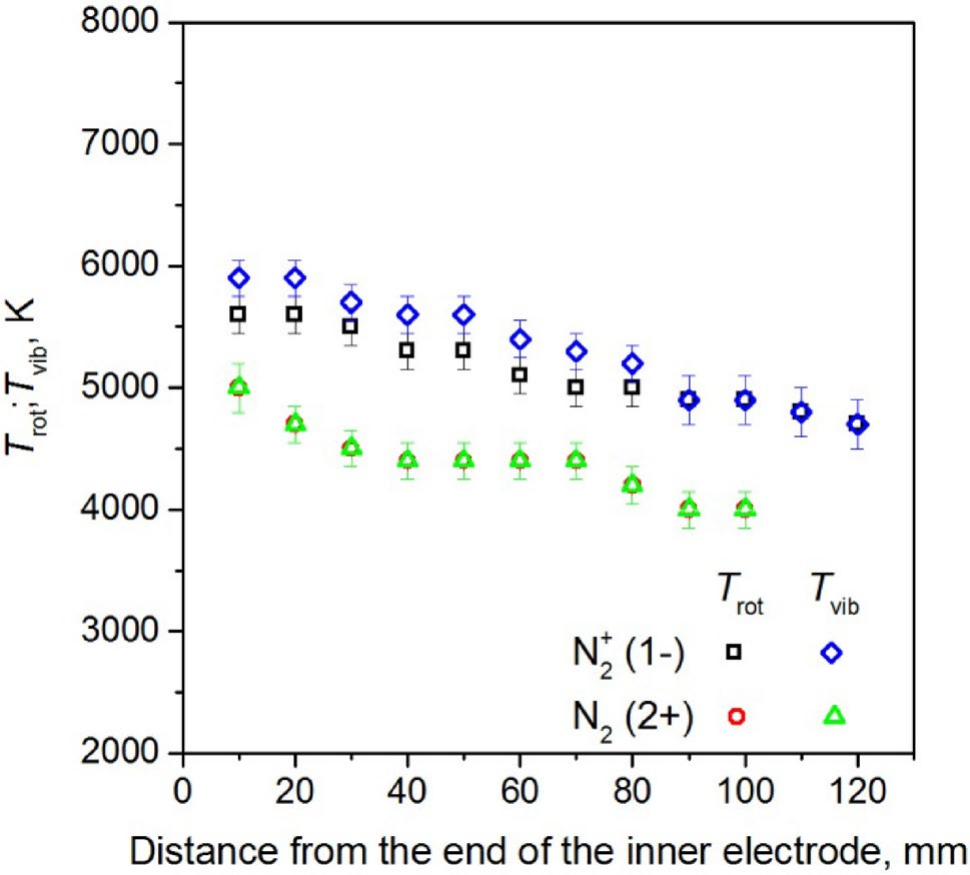
The measured values of the *T*_vib_ and *T*_rot_ for nitrogen molecules and nitrogen molecule ions as a function of distance from the end of the inner electrode, *P*_A_ = 3 kW, *Q*_N2_ = 50 Nl/min.

Figures 21, 22, and 23 illustrate the estimated values of *T*_vib_ and *T*_rot_ for nitrogen molecules and nitrogen molecule ions as a function of:

- absorbed microwave power *P*_A_, Fig. 21,
- volumetric flow rate of working gas *Q*_N2_, Fig. 22,
- distance from the end of the inner electrode, Fig. 23.

The study reveals that there is no thermodynamic equilibrium between the *T*_vib_ and *T*_rot_ temperature of the nitrogen molecule ion. However, as illustrated in Fig. 23, as the distance from the end of the inner electrode increases, the measured values of the *T*_vib_ and *T*_rot_ become closer to each other. In the case of the nitrogen molecule, the *T*_vib_ and *T*_rot_ temperatures were equal. Figure 21 shows a slight increase in the measured temperatures of N_2_ and N_2_^+^ as the microwave power absorbed by the plasma increases, while Fig. 22 a slight decrease in the temperatures is observed as the nitrogen flow rate increases. With an increase in the distance from the end of the inner electrode (Fig. 23), the measured temperatures decrease. The temperatures obtained did not exhibit significant changes with an increase in the volumetric flow rate of nitrogen *Q*_N2_, as show in Fig. 22. The *T*_vib_ and *T*_rot_ of nitrogen molecular ions varied between 4700 and 6100 K, depending on the discharge conditions, while for nitrogen molecules, these values were equal and ranged from 4000 to 5300 K.

## Summary

The new MPS allows for stable microwave discharge in nitrogen at high flow rates, up to several hundred litters per minute, under atmospheric pressure. This work describes the new MPS and its electrodynamic properties, presents the results of investigations into its energy efficiency and reports on spectroscopic studies of microwave plasma generated in nitrogen.

The use of a microwave plasma model allowed for the calculation of the electrodynamic characteristics of the MPS that were consistent with experimental results, as shown in Fig. 6. These electrodynamic characteristics were analysed to improve the construction of the MPS. The main goal of this improvement was to increase the absorption of microwave energy by the generated discharge in the MPS, ultimately improving the microwave energy transfer efficiency of the plasma source and achieving a microwave loss power of less than 3% of the incident wave power in the new MPS. Compared to the MPS before improvement, this means a two-fold decreasing the reflected microwave power.

The applied plasma model has limited capabilities in fully capturing the properties of the microwave plasma generated in the MPS. Therefore, using this model in the improvement process resulted in only a partial increase in the energy efficiency of the MPS. However, the investigations conducted have provided valuable insights into plasma behavior and have contributed to the development new energy efficient MPS.

## Data Availability

The datasets used and/or analysed during the current study available from the corresponding author on reasonable request.
